# Clinical efficacy of traditional Chinese medicine therapy for female stress urinary incontinence: a meta-analysis

**DOI:** 10.1590/1980-220X-REEUSP-2023-0153en

**Published:** 2024-02-02

**Authors:** Hui Liu, Yanan Li, Han Zheng, Yiqun Miao, Shuliang Zhao, Wenting Sun, Yuanyuan Zhang

**Affiliations:** 1 Shandong Second Medical University, School of Nursing, Weifang, Shandong Province, China.; 2 Shandong Second Medical University, College of Traditional Chinese Medicine, Weifang, Shandong Province, China.

**Keywords:** Acupuncture Therapy, Acupressure, Meta-analysis, Moxibustion, Urinary Incontinence, Terapia de Acupuntura, Acupresión, Metaanálisis, Moxibustión, Incontinencia Urinaria, Terapia com Acupuntura, Acupressão, Metanálise, Moxabustão, Incontinencia Urinaria

## Abstract

**Objectives:**

To investigate the efficacy of traditional Chinese medicine (TCM) in the treatment of female stress urinary incontinence (SUI).

**Method:**

PubMed, Cochrane, Web of Science, Embase, CNKI, Wanfang, and VIP databases were searched for articles published up to September 2022. Variables were analyzed using weighted mean difference (WMD), standardized mean difference (SMD), odds ratios (OR), and 95% confidence interval (CI).

**Results:**

Eight studies containing 744 patients were included in this study. The results demonstrate that TCM therapy had more advantages in improving the clinical outcome of SUI patients (OR = 2.90, 95%CI:1.92–4.37, P = 0.000), reducing the International Consultation on Incontinence Questionnaire Short-Form (ICIQ-SF) score (WMD = –2.41, 95%CI:–2.83– –1.98, P = 0.000), reducing 1-h urinary pad leakage urine volume (WMD = –1.86, 95%CI:–2.23– –1.49, P = 0.000) and increasing Maximum urethral closure pressure (MUCP) (SMD = 0.86, 95%CI: 0.61–1.11, P = 0.000).

**Conclusion:**

TCM therapy is effective in improving urinary incontinence symptoms, urodynamics, and quality of life in patients with SUI. This article provides a reference for the application of TCM therapy in women with urinary incontinence.

## RESUMEN

**Objetivo:**

Investigar la eficacia de la medicina tradicional china (MTC) en el tratamiento de la incontinencia urinaria de esfuerzo (IUE) femenina.

**Método:**

Se llevaron a cabo búsquedas de artículos publicados hasta septiembre de 2022 en las bases de datos PubMed, Cochrane, Web of Science, Embase, CNKI, Wanfang y VIP. Las variables se analizaron mediante la diferencia de medias ponderada (DMP), la diferencia de medias estandarizada (DME), la razón de momios (RM) y el intervalo de confianza (IC) del 95%.

**Resultados:**

Este estudio incluyó ocho investigaciones con la participación de 744 pacientes. Los resultados indican que la terapia de medicina tradicional china (MTC) presentó mejoras significativas en los resultados clínicos de pacientes con incontinencia urinaria de esfuerzo (IUE) (RM = 2.90, IC del 95%: 1.92–4.37, P = 0.000), al reducir la puntuación del Cuestionario Internacional sobre la Incontinencia y su impacto en la calidad de vida (ICIQ-SF) (DMP = –2.41, IC del 95%: –2.83– –1.98, P = 0.000), así como la disminución del volumen de orina a la 1 hora de fuga de la almohadilla urinaria (DMP = –1.86, IC del 95%: –2.23– –1.49, P = 0.000) y el aumento de la presión de cierre uretral máxima (PCUM) (DME = 0.86, IC del 95%: 0.61–1.11, P = 0.000).

**Conclusión:**

La terapia de medicina tradicional china (MTC) resulta efectiva en la mejora de los síntomas de la incontinencia urinaria, la urodinámica y la calidad de vida en pacientes con incontinencia urinaria de esfuerzo (IUE). Este artículo ofrece una referencia para la aplicación de la terapia de MTC en mujeres con incontinencia urinaria.

## RESUMO

**Objetivo:**

Investigar a eficácia da medicina tradicional chinesa (MTC) no tratamento da incontinência urinária de esforço (IUE) feminina.

**Método:**

Foram realizadas pesquisas de artigos publicados até setembro de 2022 nas bases de dados PubMed, Cochrane, Web of Science, Embase, CNKI, Wanfang e VIP. As variáveis foram analisadas por meio da diferença de média ponderada (DMP), da diferença média padronizada (DMP), da razão de chances (RC) e do intervalo de confiança (IC) de 95%.

**Resultados:**

Esta pesquisa envolveu oito estudos, contando com a participação de 744 pacientes. Os resultados indicam que a terapia da medicina tradicional chinesa (MTC) apresentou melhorias significativas nos resultados clínicos de pacientes com incontinência urinária de esforço (IUE) (RM = 2.90, IC de 95%: 1.92–4.37, P = 0.000), ao reduzir a pontuação do Questionário Internacional sobre a Incontinência Urinária – Versão Breve (ICIQ-SF) (Diferença de Média Ponderada = –2.41, IC de 95%: –2.83– –1.98, P = 0.000), assim como na diminuição do volume de urina na 1 hora de vazamento do absorvente urinário (Diferença de Média Ponderada = –1.86, IC de 95%: –2.23– –1.49, P = 0.000) e no aumento da pressão máxima de encerramento uretral (PMCU) (Diferença Média Padronizada = 0.86, IC de 95%: 0.61–1.11, P = 0.000).

**Conclusão:**

A terapia da medicina tradicional chinesa (MTC) é eficaz na melhoria dos sintomas da incontinência urinária, na urodinâmica e na qualidade de vida em pacientes com incontinência urinária de esforço (IUE). Este artigo fornece uma referência para a aplicação da terapia da MTC em mulheres com incontinência urinária.

## INTRODUCTION

Stress urinary incontinence (SUI) is the prevailing form of urinary incontinence^(1)^, with a prevalence of up to 15% among women aged 30–60 years^(2)^. SUI is a symptom of involuntary loss of urine due to effort or physical exertion (such as physical activity) or sneezing or coughing^(3)^. SUI exerts a substantial adverse influence on the physical, psychological, and social interactions of patients’ well-being^(1)^. The lack of treatment for SUI has been found to be linked with several negative outcomes, including falls and fractures, sleep disturbances, and urinary tract infections^(4)^. Moreover, as a result of the involuntary discharge of urine and prolonged urinary leakage, individuals are susceptible to experiencing adverse psychological states like anxiety, melancholy, and feelings of humiliation^(5)^.

The current treatments for SUI are primarily surgical and non-surgical. Mid-urethral suspension and retropubic vaginal suspension are surgical treatments^(6)^. However, surgical treatment is invasive and prone to complications. According to the Polish Society of Obstetricians and Gynaecologists (PSGO), non-surgical therapy is the preferred option for all women with SUI, regardless of severity^(7)^. Non-surgical therapies commonly used include lifestyle changes, medication, and pelvic floor muscle training (PFMT). There is currently no high-quality data on how PFMT can be conducted to improve outcomes for women with SUI^(8)^. Furthermore, high-quality information on the best kind, dosage, and duration of oestrogen treatment is insufficient. As a result, it is critical to investigate quick and efficient therapies for SUI. Traditional Chinese medicine (TCM) is the traditional medicine of China, and TCM has a long history of recognizing SUI. TCM has been shown in studies to be effective, simple to conduct, and have little side effects^(9, 10, 11)^. Acupuncture, moxibustion, acupressure, and TCM decoction are all methods of treating SUI in TCM. Acupuncture includes inserting needles into acupuncture points to stimulate the skin and meridians, allowing qi and blood to flow smoothly through the meridians for therapy. Moxibustion is a type of acupuncture therapy that involves the use of lighted moxa at acupuncture points or particular regions. Acupressure is a meridian-based treatment practice that uses fingers, palms, and elbows to activate acupuncture points by performing massage methods on the patient^(12)^.TCM decoction is a liquid preparation created by boiling medicinal ingredients in water. As a result, we conducted this meta-analysis to systematically evaluate the efficacy of TCM therapy in women with SUI.

## METHOD

### Patient and Public Involvement

Our study did not have patient participation.

### Systematic Search and Strategy

The Preferred Reporting Items for Systematic Reviews and Meta-Analyses (PRISMA) reporting guidelines were strictly followed when conducting this study^(13)^. The following databases were searched: PubMed, Cochrane, Web of Science, Embase, CNKI, Wanfang, and VIP. Articles published between the creation of the database and September 2022 were retrieved. The researchers independently searched the papers using the following search terms: (‘Urinary Incontinence’ OR ‘Incontinence, Urinary’) AND ((‘Medicine, Chinese Traditional’ OR ‘Traditional Chinese Medicine’) OR (‘Acupuncture Therapy’ OR ‘Acupuncture Treatment’ OR ‘Acupuncture Treatments’) OR (‘Moxibustion’ OR ‘Moxabustion’) OR (‘Acupressure’ OR ‘Shiatsu’) OR (‘Cupping Therapy’ OR ‘Cupping Treatment’) OR (‘Acupuncture Point’ OR ‘Acupuncture Points’ OR ‘Point, Acupuncture’)). Articles written in either Chinese or English will be included. All databases utilise a similar search approach.

### Eligibility Criteria

#### Inclusion Criteria

Types of Studies: Randomised controlled studies using TCM therapy to treat female SUI.

Types of participants: Women aged 18 years or older with a clear diagnosis of SUI based on the patient’s symptoms of involuntary urination caused by urgency, straining, exertion, sneezing, coughing, or physical examination.

Intervention measures: PFMT was administered to the control group. Based on the control group, the experimental group underwent TCM therapy (traditional Chinese medicine, acupuncture, moxibustion, acupressure, and cupping therapy).

#### Main Outcomes

At least one of the parameters was included in the outcome measures: Clinical Effectiveness, International Consultation on Incontinence Questionnaire Short-Form(ICIQ-SF), 1h pad test, and Maximum urethral closure pressure (MUCP).

##### The Primary Outcomes

Clinical Effectiveness: Clinical Effectiveness is assessed by the improvement of the patient’s symptoms of SUI or by the change in the volume of urine leaked by the 1-h urine pad test.

ICIQ-SF: This scale includes three dimensions: the number of urine leakage, the volume of urine leakage, and the quality of life. The overall score was 21, with higher scores indicating more severe incontinence and a greater impact on quality of life.

##### The Secondary Outcomes

1-h pad test: At the end of the test, the pads were weighed, and the weight gain of the pads showed the amount of urine leakage when compared to the baseline

Urodynamic evaluation: Urodynamics can provide objective information on bladder and pelvic floor function. The MUCP is a urodynamic summary measure used to confirm SUI.

##### Exclusion Criteria

Studies that match the following criteria will be excluded: 1) Incomplete data information in the literature; 2) Animal experiments, reviews, case reports, and conference abstracts of relevant studies.

### Data Extraction and Quality Assessment

The literature was reviewed separately by a TCM specialist and a researcher. The literature retrieved in the database was imported into the literature management software (EndNote x9) for screening. The data extracted from the literature that met the inclusion criteria included: basic information (author, article title, publication year), experimental design (number of cases, intervention measures), and outcome indicators. When the two evaluators disagree, they will negotiate and invite a third researcher with appropriate work experience to join the conversation if they are unable to achieve an agreement. All three researchers were given the same training.

According to the methodology recommended by the Cochrane Collaboration in the Cochrane Handbook for the Systematic Evaluation of Interventional Studies - Version 5.1.0, included studies should be evaluated for risk of bias in the following seven areas: random sequence generation, allocation hiding, blinding of participating researchers, blinding of result evaluation, incomplete result data, selective reporting, and other biases. Revman version 5.3 was used by two investigators to analyse all included studies for three levels of bias (high, low, and uncertain).

### Statistical Analysis

Stata 17 software was used for meta-analysis. Weighted mean differences (WMD), standardised mean differences (SMD), and 95% confidence intervals (CI) are used to compare continuous variables, whereas odds ratios (OR) are used to compare ordered data. If I^2^ < 50%, heterogeneity was insignificant and a fixed effects model was used; if I^2^ ≥ 50%, strong heterogeneity was evidenced and a random effects model was used. Furthermore, because some factors may contribute to the appearance of heterogeneity, sensitivity analysis was undertaken to rule out these studies. Egger’s test was used to assess publication bias, and funnel plots were constructed. When P < 0.05, statistical significance was indicated.

## RESULTS

### Search Results

**Figure 1:**
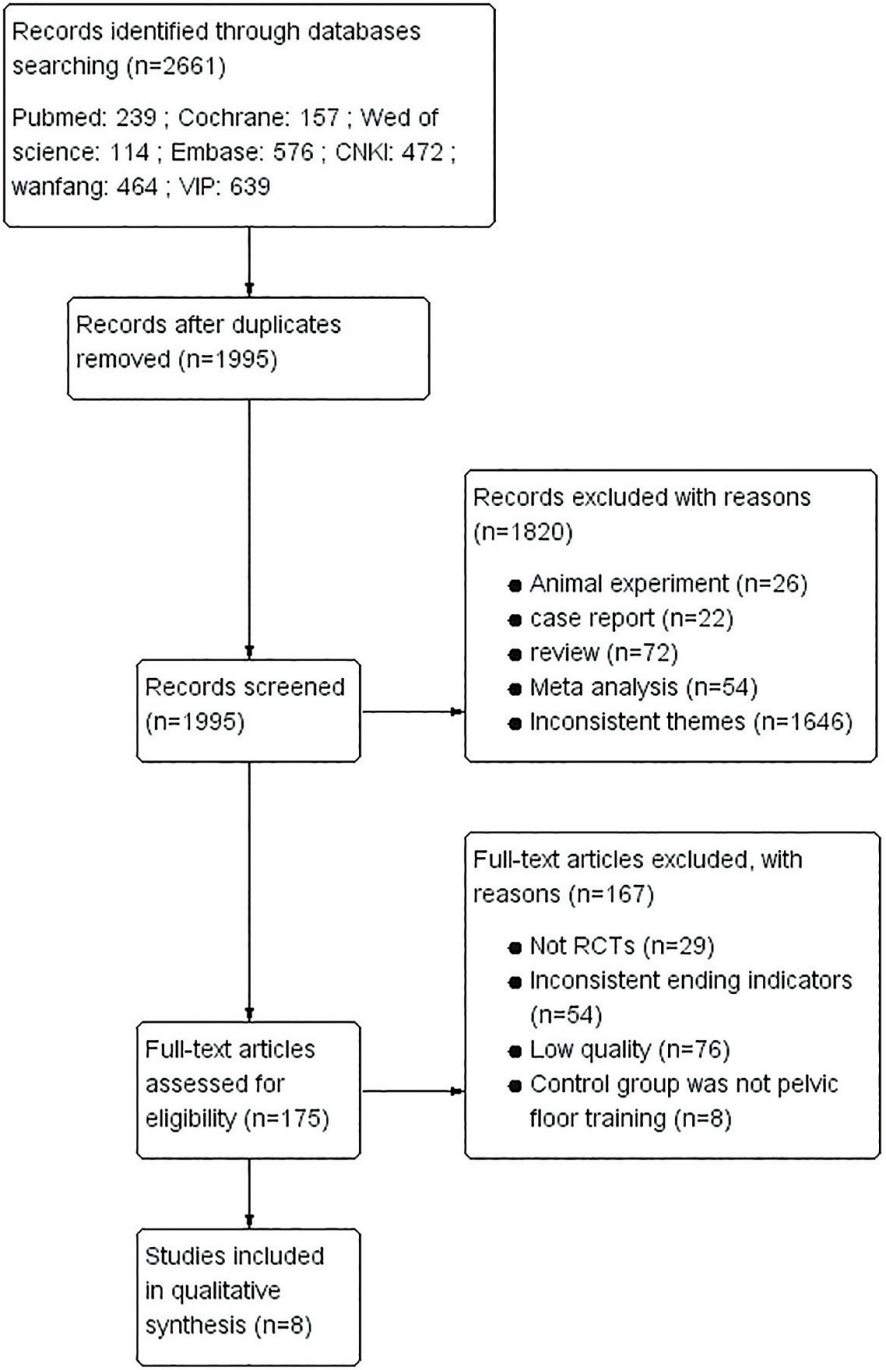
Flow chart of the study selection process.

The search approach is detailed in S1 materials. The PRISMA checklist is shown in S2. We searched a total of 2661 studies in seven databases with the following information: PubMed:239; Cochrane:157; Web of science:114; Embase:576; CNKI:472; Wanfang:464; VIP:639. After excluding duplicate data 1995 studies were retained and 1820 studies were excluded after reading the titles and abstracts. Moreover, after reading the full text of the remaining 175 studies, 29 studies were non-randomized controlled trials, 54 studies had non-compliant outcome indicators, 76 studies were of low quality and 8 studies had no PFMT in the control group. Finally, 8 studies were included. The PRISMA flow chart for choosing literature is displayed in Figure 1.

### Study Characteristics and Quality Assessment

All 8 studies included in this Meta-analysis were randomized controlled trials. The TCM therapies involved were acupuncture, moxibustion, acupressure, and TCM decoction. The control group was female patients with SUI who received PFMT. The experimental group was treated with TCM therapy based on the control group. Table 1 shows more details of the article.

**Table 1:** Characteristics of included studies – Weifang, Shandong Province, China, 2023.

Study	Year	Study type	Participants	Controls	Outcomes
Tang et al.^(14)^	2009	RCT	36 women were treated with Acupuncture, Moxibustion with tortoise shell and Pelvic floor muscle training	35 women were treated with Pelvic floor muscle training	①
Lou et al.^(15)^	2018	RCT	54 women were treated with Acupressure and Pelvic floor muscle training	53 women were treated with Pelvic floor muscle training	③
Cao et al.^(16)^	2021	RCT	33 women were treated with Warming acupuncture-moxibustion and Pelvic floor muscle training	33 women were treated with Pelvic floor muscle training	③
Deng et al.^(17)^	2020	RCT	30 women were treated with Electrothermic-needling moxibustion and Pelvic floor muscle training	30 women were treated with Pelvic floor muscle training	②
Yang et al.^(18)^	2021	RCT	75 women were treated with Moxibustion and Pelvic floor muscle training	75 women were treated with Pelvic floor muscle training	①④
Peng et al.^(19)^	2018	RCT	35 women were treated with Moxibustion and Pelvic floor muscle training	35 women were treated with Pelvic floor muscle training	①②③
Chen et al.^(20)^	2020	RCT	80 women were treated with Acupuncture and Pelvic floor muscle training	80 women were treated with Pelvic floor muscle training	①②③④
Pan et al.^(21)^	2021	RCT	30 women were treated with Tonifying Spleen and Kidney Therapy and Pelvic floor muscle training	30 women were treated with Pelvic floor muscle training	①②

Note: ① ICI-Q-SF ② 1h pad test ③ Clinical Effectiveness ④ MUCP.

One study made no mention of the generation of random sequences^(21)^. Five studies used the random number table method^(15–19)^. One study followed the block group randomization method^(20)^, and one other study used the principle of assignment with minimal imbalance index^(14)^. One studies described the allocation concealment method^(20)^. None of the studies mentioned how the blinding was performed. All studies reported outcomes for outcome indicators.

### Meta-Analysis Results

#### Clinical Effectiveness

**Figure 2:**
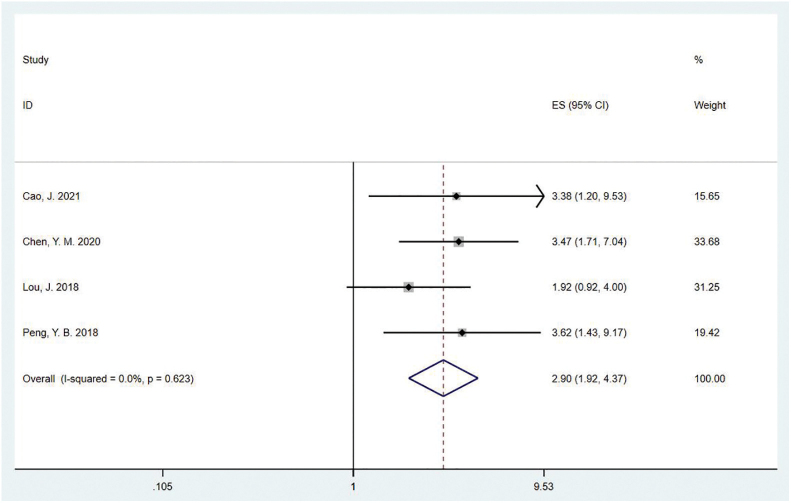
Forest plot of the effect of TCM therapy on clinical effectiveness.

Four studies with a total of 403 patients reported clinical effectiveness. A fixed-effects model was used because no statistical heterogeneity was found across studies (I^2^ = 0.0%, P = 0.623). The results showed a statistically significant difference between the two groups, (OR = 2.90, 95%CI:1.92–4.37, P = 0.000), indicating that the TCM therapy was able to relieve the patients’ urinary incontinence symptoms (Figure 2).

#### ICIQ-SF

**Figure 3:**
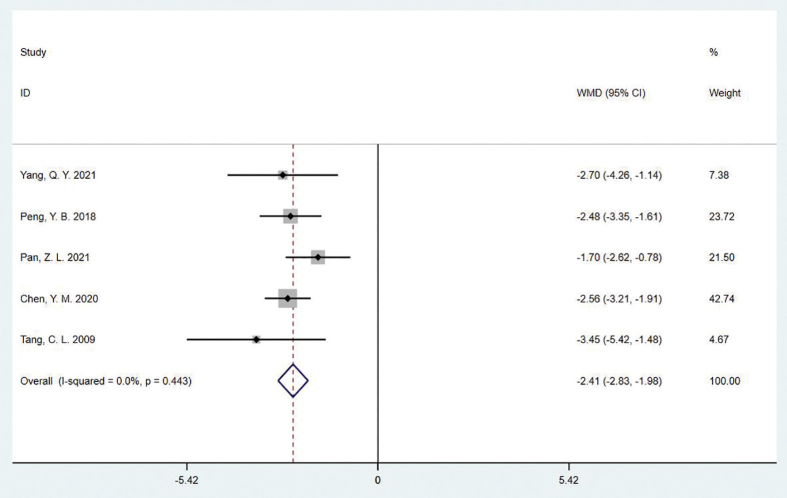
Forest plot of the effect of TCM therapy on ICIQ-SF score.

Five studies reported changes in ICIQ-SF scores relative to baseline, including 511 patients. The heterogeneity between studies was I^2^ = 0.0% and P =0.443 for the Q-test, indicating that the studies had good homogeneity. As a result, the fixed effect model was adopted in the meta-analysis. The results showed a statistically significant difference between the two groups, (WMD = −2.41, 95%CI:−2.83–−1.98, P = 0.000], the experimental group had lower scores than the control group, suggesting that TCM treatment could reduce urinary incontinence and improve the quality of life of the patients (Figure 3).

#### 1-H Pad Test

Four studies reported changes in leakage volume relative to baseline during the 1-h urine pad test in 350 patients. There was significant statistical heterogeneity among studies (I^2^ = 52.7%, P = 0.096). Sensitivity analysis showed that Deng, J. 2020 had a relatively large effect on heterogeneity. The reason for our finding of heterogeneity may be the different study populations, because the study population of Deng, J. 2020 was SUI patients with spleen-kidney Yang deficiency syndrome, and the rest of the studies were SUI patients. Heterogeneity was tested by excluding study Deng, J. 2020. The results of meta-analysis showed that TCM therapy had a significant advantage in reducing the amount of urine leakage compared to PFMT alone (WMD = –1.86, 95% CI: –2.23 – –1.49, P = 0.000), so the fixed effect model was selected.

#### MUCP

Two studies reported MUCP in women with SUI. Meta-analysis shows that TCM therapy has an advantage over other treatments in increasing MUCP in women with SUI (SMD = 0.86, 95%CI: 0.61–1.11, P = 0.000), and also showed a high degree of heterogeneity (I^2^ = 92.4%, P = 0.000), so the random effect model was selected.

### Publication Bias

Study quality and bias risk were assessed using funnel plots, and the test results showed that there was no significant risk of bias in this study. The ICI Q-SF scores in this study displayed a symmetric funnel plot.

## DISCUSSION

TCM has made significant advances to the prevention and treatment of the new coronavirus pneumonia, demonstrating increasingly distinctive value and garnering international notice and attention. However, there has been no meta-analysis of TCM in terms of SUI, and there is a lack of clear evidence-based medical evidence to support its intervention effect, so we performed a meta-analysis. Our meta-analysis showed that the addition of TCM therapy improved clinical efficacy to a greater extent, which is consistent with previous studies^(22, 23, 24)^.

SUI belongs to the category of “drowning”, “incontinence of urine” and “enuresis” in Chinese medicine^(25)^. The pathogenesis of SUI is unconsolidation of renal Qi, weakness of qi-transformation and consolidation, and loss of bladder contract; or body weakness, spleen and lung qi deficiency, and sinking of qi of middle-jiao (spleen qi deficiency and weakening of the power to maintain and lift the internal organs), resulting in urinary incontinence^(14)^. Acupuncture, moxibustion and acupressure frequently use acupuncture points in the lumbosacral area and lower abdomen, such as the foot Taiyang bladder meridian, and stimulation of this point can regulate bladder function, and promote the restoration of its urinary storage and urinary function^(26)^. From an anatomical point of view, the stimulation of specific acupuncture points can adjust the function of the internal organs, which is similar to the “somatic-sympathetic reflex” theory proposed by Western medicine^(27)^. Modern medicine believes that the sympathetic nerves innervating bladder storage come from the 11th and 12th thoracic segments and the 1st and 2nd lumbar segments, which can cause contraction of the bladder smooth muscle and the internal urethral sphincter and relaxation of the detrusor muscle to inhibit the urinary reflex^(17)^. The distribution area of acupuncture points has corresponding sympathetic nerve distribution, and stimulation of acupuncture points can cause ionic or electrical changes in the relevant nerve endings that can regulate the function of nerves, thus achieving the purpose of treating SUI^(28)^. In addition, some studies have combined conventional acupuncture with electrostimulation, and the results reveal that electroacupuncture can increase pelvic floor nerve regeneration, strengthen muscles, and alleviate urinary incontinence^(29)^. Moreover, following electroacupuncture, serum levels of succinic acid and citric acid dropped, suggesting that electroacupuncture can cure SUI by regulating the tricarboxylic acid (TCA) cycle to improve energy metabolism disorders^(30)^. Additionally, the heat generated by moxibustion can regulate the function of meridians and internal organs by stimulating acupuncture points^(31,32)^. According to certain research, the warming impact of moxibustion can enhance capillary blood flow to the bladder and pelvic floor muscles as well as heal damaged pelvic floor muscles^(18)^, thus improving SUI.

The results showed that TCM decoctions enhanced the clinical cure rate in women with SUI, and several published studies support this result^(33,34)^. According to TCM, a weak spleen and kidneys cannot control the urethra, which leads to SUI. The TCM decoctions involved in this study was Jianpiyishen decoction, and the main medicinal materials were Astragalus, Ginseng, Licorice, Cimicifuga, Bupleurum, Atractylodes. Jianpiyishen decoction can enhance the functions of the spleen and kidneys to achieve therapeutic effects. Studies on the chemical composition of TCM have revealed that supramolecular structures in TCM decoctions can act as drug carriers to facilitate the absorption and distribution of drug components and may have better biological activity than single active ingredients or their physical mixtures^(35)^. Some studies have proved that the level of vasoactive intestinal peptide (VIP) and pituitary adenylate cyclase-activating polypeptide (PACAP) in SUI patients’ vaginal tissues are reduced, and PACAP and VIP have the effect of vasodilating and diastolic smooth muscle, and TCM decoction can regulate the level of PACAP and VIP and play an effect of improving SUI symptoms^(34,36)^.

SUI is not life-threatening, but its symptoms affect patients’ daily life. The impact of SUI on the quality of life has not been fully understood, and relevant studies have found that effective treatment of SUI can improve women’s quality of life. Our study evaluated the distress of SUI patients by ICIQ-SF scale, and the results showed that the experimental group scored lower than the control group after treatment, suggesting that TCM therapy has the potential to enhance the quality of life among individuals with SUI.

No serious adverse reactions were reported in this study. Women with SUI who received acupuncture point compresses experienced irritation and discomfort; women with SUI who applied TCM decoctions experienced fatigue and headache, which improved with symptomatic treatment.

### Limitation

We hope that our study will provide stronger evidence for the application of TCM therapy in the treatment of female SUI. Nonetheless, this study has some limitations. For starters, the small total number of included studies and the small sample size of some interventions may reduce statistical efficacy. Second, the lack of uniform requirements for age, race and dose, acupoint selection, and duration of interventions in the included subjects may be the main reason for heterogeneity. Third, there is a lack of data information on the safety of Chinese medicine, and further high-quality, large-sample, multicenter studies are required for further confirmation.

## CONCLUSION

According to the findings of this study, TCM therapy dramatically improved the clinical outcome of women with SUI, lowering incontinence symptoms and enhancing quality of life. This demonstrates the advantages of TCM in the treatment of SUI. Meanwhile, this study provides Chinese medicine ideas on how to better treat female SUI on the one hand, and on the other hand, healthcare professionals can choose the appropriate rehabilitation treatment based on the actual needs of women, implement precise and personalized care to improve female SUI and improve the quality of life. However, there is a scarcity of high-quality research and therefore more high-quality studies need to be included for further validation.
